# A comprehensive clinical and laboratory evaluation of 224 patients with persistent symptoms attributed to presumed tick-bite exposure

**DOI:** 10.1371/journal.pone.0247384

**Published:** 2021-03-18

**Authors:** Kenneth Nilsson, Elisabet Skoog, Viktor Jones, Lisa Labbé Sandelin, Christina Björling, Ester Fridenström, Marie Edvinsson, Andreas Mårtensson, Björn Olsen

**Affiliations:** 1 Department of Medical Sciences, Section of Infectious Diseases, Uppsala University, Uppsala, Sweden; 2 Department of Medical Sciences, Section of Clinical Microbiology, Uppsala University, Uppsala, Sweden; 3 Department of Medical Sciences, Zoonosis Science Centre, Uppsala University, Uppsala, Sweden; 4 Department of Communicable Diseases and Disease Control, Kalmar County Hospital, Kalmar, Sweden; 5 Department of Women’s and Children’s Health, International Maternal and Child Health, Uppsala University, Uppsala, Sweden; Tufts University Cummings School of Veterinary Medicine, UNITED STATES

## Abstract

**Background:**

Persistent symptoms attributed to presumed tick-bite exposure constitute an unresolved medical controversy. We evaluated whether Swedish adults who met the criteria for post-treatment Lyme disease syndrome (PTLDS) exhibited characteristics distinguishable from adults who did not, but who displayed similar symptoms and disease course after suspected previous tick-bite infection (TBI).

**Methods and findings:**

During 2015–2018, 255 patients–referred to the Centre for Vector-borne Infections, Uppsala University Hospital, Sweden with symptoms lasting longer than six months–were recruited. Of this group, 224 completed the study. Each patient was examined by an infectious disease specialist and, besides a full medical history, underwent a panel of blood and cerebrospinal fluid laboratory tests including hematological, biochemical, microbiological and immunological analyses, and the RAND-36 scale to measure quality of life. For analysis purposes, patients were divided into five subgroups, of which one represented PTLDS. According to serological results indicating TBI and documented/ reported objective signs of Lyme disease, 85 (38%) patients fulfilled the criteria for PTLDS and were compared with the other 139 (62%) serologically classified patients. In the PTLDS group, erythema chronicum migrans (ECM) was documented/reported in 86% of patients, previous neuroborreliosis in 15%, and acrodermatitis chronica atroficans (ACA) in 3.5%. However, there were no significant differences regarding symptoms, laboratory results or disease course between patients with PTLDS and those without laboratory evidence of *Borrelia* exposition. Most reported symptoms were fatigue-related (70%), musculoskeletal (79%), neurological (82%) and neurocognitive (57%). Tick bites were recalled by 74%. The RAND-36 score was significantly below that of the general Swedish population. Signs of immunological/inflammatory reactivity with myositis antibodies were detected in 20% of patients, fibrinogen levels were moderately increased in 21% and elevated rheumatoid factor in 6%.

**Conclusions:**

The PTLDS group did not differ exclusively in any respect from the other subgroups, which either lacked previously documented/reported evidence of borreliosis or even lacked detectable serological signs of exposure to Lyme disease. The results suggest that symptoms often categorized as Chronic-Lyme-Disease (CLD) in the general debate, cannot be uniquely linked to Lyme disease. However, approximately 20% of the total group of patients showed signs of autoimmunity. Further studies are needed to elucidate the underlying causes and mechanisms of PTLDS and there is reason to consider a multifactorial approach.

## Introduction

Persistent symptoms after tick-bite exposure constitute a medical controversy regarding causality, management and treatment [1]. In Europe, Lyme disease (LD) is commonly associated with *Borrelia burgdorferi*, *B*. *garinii* or *B*. *afzelii* and diagnosed using clinical assessment along with serological testing [2]. Other genospecies (*B*. *bavariensis*, *B*. *lusitaniae*, *B*. *spielmanii*, *B*. *valaisiana*) have been linked to clinical manifestations and have been identified in various clinical samples and in questing ticks toa lesser extent [3]. Besides objective manifestations, e.g., ECM, or late stages with neurologic, cardiac, skin or joint manifestations (i.e., acrodermatitis chronica atroficans [ACA] and arthritis), some patients experience lingering symptoms of unknown etiology, such as fatigue, sleep disorders, malaise, headache, musculoskeletal pain, depression and impaired cognitive ability [4,5]. There is an increased awareness of LD among the public and in the media, with an inclination toward considering persistent discomfort of unknown origin to be an expression of chronic LD (CLD) or variants thereof. Concepts presuming persistent infection–such as the sometimes mild and self-limiting "Late persistent LD" (LPLD) or "post-treatment LD syndrome" (PTLDS), with a duration of more than six months–have been suggested diagnoses for patients, although the effect of antibiotics is questionable and rarely supported by objective clinical and/or laboratory findings [1,4,6–10]. Results of unvalidated in-house tests or non-evidence-based interpretations of standard serological tests are sometimes used to support the use of prolonged antibiotic treatment [11,12]. The burden and impact of other TBI’s (e.g., bartonellosis, anaplasmosis, babesiosis, rickettsiosis, neoehrlichiosis and manifestations, in the form of single or co-infections along with Lyme disease (LD) are incompletely understood [13–17]. In the present study, we have focused on tick-borne co-infections. Many other common infectious agents have also been proposed to be involved in persistent illness, e.g., CMV, HH7, ParvoB19, enterovirus, Candida, mycoplasmata, *Helicobacter pylori*, Campylobacter, and toxoplasma. However, their role in PTLDS remains equally speculative or incompletely understood [18]. It has also been suggested that prolonged persistent symptoms after LD may be attributed to a “central sensitization syndrome,” in which there is increased sensitivity to sensory signals in the brain. The sensitization may be a result of infectious or non-infectious exposures, but symptoms may continue long after spirochetal eradication [19].

In 2015, the Center for Vector-borne Infections (CVI) was established at Uppsala University Hospital, Sweden, in collaboration between the Sections of Infectious Diseases and Clinical Microbiology. The aim was to evaluate patients with persistent symptoms after known or suspected TBI, where CLD, LPLD or PTLDS had at some point been discussed as a conceivable cause. Using standardized protocols, the patient’s symptoms and laboratory data were assessed to determine whether they could be related to any of the TBIs endemic to Sweden. The present paper reports on a study of 224 patients with more than six months’ history of symptoms after previous known or presumed tick exposure, including their laboratory evidence of co-infection.

## Material and methods

### Study population

Between October 2015 and December 2018, 255 patients were prospectively enrolled after referral to CVI by policlinics or hospitals from central and southern Sweden. To be considered for examination, patients should fulfill at least four of seven predefined inclusion criteria, of which symptom duration > 6 months was mandatory. The other six criteria were ≥18 years of age with suspicion of previous TBI based on: (a) previous tick exposure; (b) symptoms consistent with TBI; (c) laboratory findings (i.e., microbiological results), (d) previous treatments for TBI and/or (e) laboratory or clinical suspicion of co-infection with other TBI. All patients provided written informed consent prior to study enrollment. At a later stage, after completion of the investigation, patients were divided into two Groups: those who met the criteria for PTLDS and those who did not. The latter group was divided into four subgroups based on serological results. The classification into serological groups is described below in the results section. The criteria used for PTLDS were in accordance with the Swiss and US case definitions of PTLDS, i.e., a clinically and/or laboratory documented episode of LD that, despite appropriate antibiotic treatment, leads within 6 months post-treatment to a constellation of disabling symptoms consisting of at least one of the following: fatigue, widespread musculoskeletal pain or cognitive problems [10]. The course of PTLDS may be continuous or relapsing, but it must span a period of at least 6 months. Patients categorized as having PTLDS had been previously treated for LD based on objective signs (ECM, neuroborreliosis or ACA). Seropositivity in combination with suspicion of LD, but without objective signs, was not considered sufficient to be assessed as PTLDS. However, seronegative patients presenting objective signs, such as erythema migrans, were included if the other criteria were met. Patients with other diagnoses that undeniably explained their symptoms were excluded from the study.

#### Ethics statement

The study, which was affiliated with the Department of Medical Sciences, Uppsala University, Sweden was reviewed and approved by the Swedish Ethical Review Authority, Uppsala, Sweden (reg. no. 2015/249).

### General outcome measures

Each patient was interviewed and physically examined by an infectious disease specialist. All patients answered a standardized questionnaire with 66 items covering physical and mental symptoms, their severity and duration, exposure to ticks and previous treatments with antibiotics, cortisone or anti-inflammatory medications.

The questionnaire RAND-36 was used to map individual basic health factors and to compare these factors to the burden of disease. It consists of 36 items covering eight multifunctional scales: physical function (PF), role-physical (RP), body pain (BP), general health (GH), vitality (VT), social function (SF) role-emotional (RE) and mental health (MH). Each scale is converted directly to a scale from 0 to 100, under the assumption that each question carries the same weight. The lower the score, the more severe the disability [20]. Normative mean SF-36 scores from a previous study on the general Swedish population (n = 8930; 15–93 years of age) are used as comparison [21]. The algorithms for BP and GH differ somewhat between the RAND-36 and SF-36, but are negligible at the group level, which allows reliable comparisons of results.

### Statistical analysis

The study was designed to be exploratory, with given inclusion criteria and an unpredictable outcome. Thus, no primary analysis was set and no power calculation was performed. Instead, an arbitrary target of 250 patients was set based on the number of patients who could be included during the initial years of the study. After 3 years, that number had been achieved and the analysis of collected data began. Five groups were formed (Groups 0 = PTLDS, and Groups 1–4) based on serological outcome, and all comparisons have been made between these five groups. Baseline characteristics reported symptoms and laboratory results were summarized using frequencies for categorical variables and median and range for continuous variables. For tests of between-group differences, Fishers exact test was used for categorical variables and the Kruskal–Wallis test for continuous variables. Kruskal-Wallis test was also used to test the between-group differences regarding the RAND 36 questionnaire. All statistical tests were 2-tailed, and the significance level was set at p<0.05. All p-values were adjusted for multiple comparisons using Benjamini and Yekutieli’s method [22]. The R version 3.5.0 statistical software (www.r-project.org) was used for the analyses. The data used for statistical tests and the results can be found in S1–S4 Files.

### Laboratory investigation

All patients underwent a standardized panel of blood and cerebrospinal fluid (CSF) laboratory tests to capture evidence of infection and/or inflammation, including hematological, biochemical, immunological and microbiological analyses, where the majority of tests were performed as part of routine diagnostics at the Academic University Hospital, Uppsala, Sweden. Lumbar puncture was performed for analysis of cells (10^6^/L), albumin (mg/L), lactate (mmol/L), glucose ratio (>0,5), protein fractions (mg/L), glial fibrillary acidic protein and CXCL-13 (ng/L) in all patients who did not have contraindications, such as having undergone back surgery, having previously reported problems with severe headache after lumbar puncture or having asked to refrain from this examination for other reasons. The hematological and biochemical panel included hemoglobin (g/L), sedimentation ratio (mm), C-reactive protein (mg/L), white blood cell count (10^9^/L), platelet cell count (10^9^/L), blood glucose (mmol/L), sodium (mmol/L), potassium (mmol/L), calcium (mmol/L), magnesium (mmol/L), creatinine (μmol/L), blood-cell-counts (10^9^/L), protein fraction electrophoresis, aspartate aminotransferase (μkat/L), alanine (μkat/L), alkaline phospatase (μkat/L), cobalmin (pmol/L), thyroid-stimulating hormone (mU/L), thriiodothyronine free (pmol/L), thyroxine free (pmol/L) and angiotensin converting enzyme (U/L). Of the immunological assays, besides rheumatoid factor (IU/mL), CCP-ab (IgG) and HLA typing (HLA DRB-1), antinuclear antibody testing ANA (IgG) was performed with indirect immune fluorescence on HEp-2 cells (ImmonoConcepts, Sacramento, CA, USA), with a screening dilution of 1:200, corresponding to a 94% diagnostic specificity when investigating 200 healthy blood donors. Evaluations of individual ANA specificities were conducted using a 14-plex addressable laser bead immunoassay (FIDIS Connective, Theradiag, Beaubourg, France), investigating autoantibodies against SSA/Ro52, SSA/Ro60, SSB, Sm, U1RNP, the Sm/U1RNP complex, double stranded DNA, histones, ribosomal P antigen, proliferating cell nuclear antigen (PCNA), Jo-1, Scl-70, CENP-B and PM-Scl. Myositis-specific antibodies (MSA) and myositis-associated antibodies (MAA) were investigated using the Inflammatory Myopathies 16 antigen line immunoassay (Euroimmun, Lübeck, Germany), including separate determination of autoantibodies against Mi-2 alpha chain, Mi-2 beta chain, TIF1g, MDA5, NXP2, SAE1, Ku, PM-Scl100 kD, PM-Scl 75 kD, Jo-1, SRP, PL-7, PL-12, EJ, OJ and SSA/Ro52. All assays were performed according to the manufacturers’ instructions.

The microbiological panel tested antibodies in serum and CSF to *Borrelia* spp., including *B*. *burgdorferi*, *B afzelii* and *B*. *garinii* (Euroimmun®, Lübeck, Germany), and Euroline-Western blot analysis (Euroimmun®, Lübeck, Germany) was performed to evaluate the specificity of the reactivity. The Euroline-WB assay uses highly purified native or recombinant antigens that are printed as lines onto nitrocellulose strips. The Euroline-IgG strips contain antigen against p17, p19, p21, OspC (p25), p30, OspA (p31), BmpA (p39), p83 and VlsE and the Euroline-IgM strips against p17, p19, p21, OspC (p25), p28, p30). The band reactivity is compared and interpreted by the manufacturer’s software in accordance with the standardized algorithm and presented in separate result sheets as positive or negative. Assessment of the serological results was based on the results for both ELISA and WB. A negative ELISA with a low-positive WB was judged to be negative, in accordance with current interpretation criteria. Similarly, false positivity for IgM in ELISA was excluded. The conformity between ELISA and WB was generally very good, and WB generally confirmed the ELISA results. In serum, screening was performed for antibodies against *Anaplasma phagocytophilum* (Focus Diagnostics®, Cypress, CA, USA), *Bartonella henselae* and *B*. *quintana* (Euroimmun®, Lübeck, Germany), *Rickettsia* spp. [14] and TBEV (Immunozym FSME IgM and IgG, respectively, Progen Biotechnik GmbH, Germany). The serologic tests (IFA) for *Babesia divergens* and *B*.*microti* were performed at the Public Health Agency of Sweden, Solna, Stockholm and National Institute for Public Health and the Environment (RIVM), Bilthoven, the Netherlands. Serology (IFA) and PCR of *Rickettsia* spp. in CSF were performed at Academic University Hospital, and PCR of *Candidatus* Neoehrlichia mikurensis in blood at Sahlgrenska University Hospital, Gothenburg, Sweden [17,23].

### Overall assessment of outcomes

Each patient’s overall result was assessed in plenary by the research group. Thereafter, a consensus statement, in accordance with the study’s approval, was sent to the referring physician for information and use in patient management. A determination was made as to whether any additional sampling, antibiotic treatment or referral of the patient for further investigation was called for.

## Results

### Study population, characteristics and background data

A total of 255 patients was assessed, of whom 31 were excluded because they either declined sampling or ultimately could not participate. The remaining 224 patients were included in the analysis (103 men [46%] and 121 women [54%]). The overall median age was 55 years (range 18–92), 58 years (range 19–81) and 51 years (range 18–92) for men and women, respectively. The 224 patients were divided into five subgroups. Group 0 represented 85 patients who met the criteria for PTLDS. Group 1 and 2 were seropositive for *Borrelia burgdorferi* s.l. (Bb s.l.), but lacked documented/reported objective signs for LD. Group 1 (n = 31) included patients with antibodies only to *Bb* s.l.; Group 2 (n = 40) included patients with antibodies to *Bb* s.l. and any of the other TBIs.

Group 3 and 4 were both seronegative for Bb s.l.. Group 3 (n = 32) included patients with antibodies to any of the other TBIs, but not to *Bb* s.l., and Group 4 (n = 36) patients with no antibodies to *Borrelia* s.l. or TBIs. The baseline demographic, epidemiological and clinical data, merged for the entire cohort and for the respective groups, are summarized in Tables 1–3. Regarding the background data (Table 1), the daily activities, which did not differ between the groups, were distributed as follows: working full-time (39%), full-time sick leave (21%), part-time sick leave (10%), retirement (23%) and other (7%). Documented/reported objective signs such as neuroborreliosis, ECM and ACA were significantly (*p* <0.05) more common in the PTLDS group and in Group 1. Moreover, the PTLDS patients and patients in Group 1 had previously received antibiotic treatment at a significantly (*p* < 0.05) higher frequency, had experienced more treatment episodes and a greater number/variety of antibiotics than the other groups. In addition, there were no significant between-group differences in duration of symptoms, previous tick bite, ongoing sick leave, or daily activities. As a whole, the PTLDS group showed the same presentation of clinical and laboratory results as the entire group of patients, regardless of previous exposure to TBI.

**Table 1:** Characteristics and background data ✫.

	Group 0 PTLDS (no. = 85)	Group 1 Bb s.l. POS other TBI NEG (no. = 31)	Group 2 Bb s.l. POS other TBI POS (no. = 40)	Group 3 Bb s.l. NEG other TBI POS (no. = 32)	Group 4 Bb s.l. NEG other TBI NEG (no. = 36)	TOTAL 0–4 (no. = 224)
Age—*year median (range)*	58 (20–92)	55 (26–76)	59 (18–83)	45 (19–77)	44 (19–68)	55 (18–92)
Male/Female—*no*. *(%)*	40/45 (47/53)	17/14 (55/45)	19/21 (48/52)	12/20 (38/62)	15/21 (42/58)	103/121 (46/54)
Patients with symptoms of more than 1 year—*no*. *(%)*	74 (87)	25 (81)	34 (85)	26 (81)	31 (85)	190 (84)
Symptom duration—*median (range)*	4 (1–26)	4 (1–29)	3 (1–13)	5 (1–40)	5 (1–34)	4 (1–40)
Patients that recalled tick bite—*no*. *(%)*	64 (75)	25 (81)	26 (65)	23 (72)	27 (75)	165 (74)
Patients with documented erythema migrans	73 (86)*	0 (0)	0 (0)	0 (0)	0 (0)	73 (33)
Patients with documented neuroborreliosis	13 (15)*	0 (0)	0 (0)	0 (0)	0 (0)	13 (6)
Patients with documented acrodermatitis atroficans	3 (3.5)	0 (0)	0 (0)	0 (0)	0 (0)	3 (1)
No. of reported symptoms—*median (range)*	8 (3–15)	7.5 (5–16)	7 (2–18)	8.5 (3–15)	8 (4–14)	8 (2–18)
Previous antibiotic treatment for Lyme disease—*no*. *(%)*	82 (96)*	27 (87)	30 (75)	15 (47)	22 (61)	176 (79)
Duration—*days median (range)*	14 (10–165)	14 (10–21)	14 (10–90)	10 (10–350)	10 (10–90)	14 (10–350)
IV antibiotics—*no*. *(%)*	8 (9)	3 (10)	4 (10)	1 (3)	2 (6)	18 (8)
No. of antibiotic treatments—*median (range)*	2 (0–6)*	1 (0–4)*	1 (0–3)	1 (0–6)	1 (0–5)	1 (0–6)
No. of different antibiotics used—*median (range)*	1 (0–3)*	1 (0–2)*	1 (0–2)	1 (0–4)	1 (0–2)	1 (0–4)
Previously investigated at a clinic abroad—no. (%)	10 (12)	3 (10)	5 (12·5)	3 (9)	8 (22)	29 (13)
Previously treated at a clinic abroad—*no*. *(%)*	7 (8)	2 (6)	3 (7·5)	3 (9)	2 (6)	17 (8)
Ongoing sick-leave due to current symptoms—*no*. *(%)*	30 (35)	10 (32)	10 (25)	8 (25)	13 (36)	71 (32)
At work (full time or part time)—*no*. *(%)*	35 (41)	20 (65)	20 (50)	20 (63)	22 (61)	117 (52)
Proposed antibiotic treatments after CVI assessment—*no*. *(%)*	5 (6)	2 (6)	4 (10)	3 (9)	0 (0)	14 (6)
Borrelia—*no*. *(%)*	2 (2.5)	2 (6)	2 (5)	0 (0)	0 (0)	6 (3)
Rickettsia—*no*. *(%)*	0 (0)	0 (0)	0 (0)	1 (3)	0 (0)	1 (0.4)
Anaplasma—*no*. *(%)*	1 (1)	0 (0)	1 (2.5)	1 (3)	0 (0)	3 (1.3)
Bartonella—*no*. *(%)*	0 (0)	0 (0)	1 (2.5)	0 (0)	0 (0)	1 (0.4)
C. Neoehrlichia—*no*. *(%)*	2 (2)	0 (0)	0 (0)	1 (3)	0 (0)	3 (1.3)
Fulfilling suggested critereria for PTLDS—*no*. *(%)*	85/85 (100)	0/31 (0)	0/40 (0)	0/32 (0)	0/36 (0)	85/224 (38)

✫ Bb s.l. denotes *Borrelia burgdorferi* sensu latu, TBI tick borne infection, N total number, no. number, PTLDS post-treatment LD syndrome.

* Statistically significant difference at p<0.05 level compared to the groups with no *.

**Table 2:** Reported symptoms ✫.

	Group 0* PTLDS (no. = 85)	Group 1 Bb s.l. POS other TBI NEG (no. = 31)	Group 2 Bb s.l. POS other TBI POS (no. = 40)	Group 3 Bb s.l. NEG others TBI POS (no. = 32)	Group 4 Bb s.l. NEG others TBI NEG (no. = 36)	TOTAL (0–4) (no. = 224)
General symptoms—*no*. *(%)*						
Fatigue	56 (66)	24 (77)	26 (65)	24 (75)	27 (75)	157 (70)
Sleep disturbances	31 (36)	12 (39)	15 (38)	14 (44)	14 (39)	86 (38)
Reduced endurance	28 (33)	7 (23)	7 (18)	10 (31)	14 (39)	66 (29)
Any of the symptoms above	65 (76)	27 (87)	33 (83)	28 (88)	30 (83)	183 (82)
Musculosceletal symptoms—*no*. *(%)*		* *	* *	* *	* *	* *
Back ache	30 (35)	16 (52)	14 (35)	13 (41)	24 (67)	97 (43)
Muscle pain	30 (35)	12 (39)	20 (50)	14 (44)	10 (28)	86 (38)
Stiffness in muscles/joints	24 (28)	10 (32)	14 (35)	11 (34)	10 (28)	69 (31)
Muscle weakness	12 (14)	2 (6)	8 (20)	8 (25)	9 (25)	39 (17)
Tics	10 (12)	2 (6)	3 (8)	6 (19)	3 (8)	24 (11)
Any of the symptoms above	66 (78)	24 (77)	32 (80)	26 (81)	30 (83)	178 (79)
Neurological symptoms—*no*. *(%)*						
Numbness	19 (22)	8 (26)	6 (15)	9 (28)	8 (22)	50 (22)
Paresthesia	23 (27)	7 (23)	15 (38)	4 (13)	8 (22)	57 (25)
Nerve pain	14 (16)	3 (10)	7 (18)	2 (6)	3 (8)	29 (13)
Facial palsy	10 (12)	4 (13)	3 (8)	2 (6)	2 (6)	21 (9)
Abducens palsy	0 (0)	1 (3)	2 (5)	0 (0)	0 (0)	3 (1)
Dizziness/balance disorder	32 (38)	15 (48)	11 (28)	11 (34)	13 (36)	82 (37)
Vision disorders	13 (15)	6 (19)	9 (23)	8 (25)	5 (14)	41 (18)
Hearing disorder	12 (14)	5 (16)	5 (13)	5 (16)	2 (6)	29 (13)
Headache	33 (39)	13 (42)	18 (45)	12 (38)	16 (44)	92 (41)
Any of the symptoms above	71 (84)	25 (81)	34 (85)	26 (81)	28 (78)	184 (82)
Neurocognitive symptoms—*no*. *(%)*	* *	* *	* *	* *	* *	* *
Hard to find words	13 (15)	2 (6)	4 (10)	4 (13)	2 (6)	25 (11)
Changed walking pattern	7 (8)	0 (0)	1 (2.5)	1 (3)	1 (3)	10 (4)
Anergy/reduced commitment	0 (0)	0 (0)	0 (0)	0 (0)	4 (11)	4 (2)
Reduced memory	23 (27)	9 (29)	5 (13)	9 (28)	10 (28)	56 (25)
Impaired concentration	20 (24)	6 (19)	10 (25)	10 (31)	14 (39)	60 (27)
Depressed mood	24 (28)	8 (26)	8 (20)	9 (28)	8 (22)	57 (25)
Mood swings	2 (2)	1 (3)	1 (2.5)	2 (6)	0 (0)	6 (3)
Anxiety	1 (1)	0 (0)	2 (5)	1 (3)	5 (14)	9 (4)
Any of the symptoms above	50 (59)	17 (55)	19 (48)	19 (59)	23 (64)	128 (57)
Pains in joints—*no*. *(%)*	* *	* *	* *	* *	* *	* *
Fingers/toes	17 (20)	11 (35)	7 (18)	8 (25)	11 (31)	54 (24)
Wrists/ankles	18 (20)	8 (26)	10 (25)	6 (19)	7 (19)	49 (22)
Knees/elbows	25 (29)	7 (23)	8 (20)	6 (19)	11 (31)	57 (25)
Hips/shoulders	14 (16)	3 (10)	6 (15)	5 (16)	7 (19)	35 (16)
Any of the symptoms above	45 (53)	16 (52)	18 (45)	20 (63)	13 (36)	112 (50)
Other symptoms noted—*no*. *(%)*	* *	* *	* *	* *	* *	* *
Respiratory	20 (24)	5 (16)	3 (8)	6 (19)	3 (8)	37 (17)
Cardiovascular	21 (25)	6 (19)	11 (28)	13 (41)	4 (11)	55 (25)
Gastrointestinal	26 (31)	6(19)	13 (33)	14 (44)	11 (31)	70 (31)
Urinary tract	16 (19)	5 (16)	6 (15)	9 (28)	3 (8)	39 (17)
Endocrinological	9 (11)	4 (13)	4 (10)	3 (9)	8 (22)	28 (13)
Skin	20 (24)	2 (6)	9 (23)	5 (16)	6 (17)	42 (19)
Allergic	7 (8)	1 (3)	3 (8)	4 (13)	7 (19)	22 (10)
Fever episode/-s	15 (18)	10 (32)	5 (13)	3 (9)	8 (22)	41 (18)
Any of the symptoms above	62 (73)	21 (68)	28 (70)	27 (84)	30 (83)	168 (75)

✫ Bb s.l.denotes *Borrelia burgdorferi* sensu latu, TBI tick borne infection, N total number, no. number, PTLDS post-treatment LD syndrome.

* No statistically significant differences were found when comparing the groups.

**Table 3:** Laboratory findings ✫.

	Group 0* PTLDS (no. = 85)	Group 1 Bb s.l. POS other TBI NEG (no. = 31)	Group 2 Bb s.l. POS other TBI POS (no. = 40)	Group 3 Bb s.l. NEG other TBI POS (no. = 32)	Group 4 Bb s.l. NEG other TBI N (no. = 36)	TOTAL 1–4 (no. = 224)
Biochemical analyses—*no*. *NR/tested (%)*						
Haemoglobin/s (120–160 g/L)	80/85 (94)	30/31 (97)	35/40 (88)	30/32 (94)	29/35 (83)	204/223 (91)
Sedimentation ratio/s (< 15 mm)	73/85 (86)	28/31 (90)	31/40 (78)	26/32 (81)	29/35 (83)	187/223 (84)
C-reactive protein/s (< 5 mg/L)	81/85 (95)	28/31 (90)	36/40 (90)	30/32 (94)	32/34 (94)	207/222 (93)
White blood cell count/s (3.5–9 x10^9^/L)	78/85 (92)	28/31 (90)	38/40 (95)	29/32 (91)	32/36 (89)	205/224 (92)
Platelet cell count/s (150–350 x 10^9^/L),	82/85 (96)	30/31 (97)	37/39 (95)	31/32 (97)	33/34 (97)	213/221 (96)
Fibrinogen (< 4.2 g/L)	65/85 (76)	30/31 (97)	30/40 (75)	25/32 (78)	27/36 (75)	177/224 (79)
Blood glucose/s (4–6 mmol/L)	50/85 (59)	22/31 (71)	25/39 (64)	21/32 (66)	22/35 (63)	140/222 (63)
Sodium/s (137–145 mmol/L)	85/85 (100)	31/31 (100)	39/40 (98)	32/32 (100)	35/36 (97)	222/224 (99)
Potassium/s (3.5–5 mmol/L)	82/85 (96)	30/31 (97)	40/40 (100)	30/32 (94)	36/36 (100)	218/224 (97)
Calcium/s (2.15–2.5 mmol/L)	74/85 (87)	29/31 (94)	38/40 (95)	30/32 (94)	35/35 (100)	206/223 (92)
Magnesium/s (0.7–0.95 mmol/L)	85/85 (100)	29/31 (94)	39/40 (98)	32/32 (100)	35/35 (100)	220/223 (99)
Creatinine/s (45–90 μmol/L)	83/85 (98)	29/31 (94)	34/40 (85)	26/32 (81)	35/35 (100)	207/223 (93)
ALAT/s (0.15–1.1 μkat/L)	84/85 (99)	31/31 (100)	37/40 (93)	32/32 (100)	33/35 (94)	217/223 (97)
Alkaline phospatase/s (0.6–1.8 μkat/L)	80/85 (94)	31/31 (100)	39/40 (98)	31/32 (97)	34/35 (97)	215/223 (96)
Cobalamin/s (120–700 pmol/L)	72/85 (85)	29/31 (94)	36/40 (90)	25/32 (78)	29/35 (83)	191/223 (86)
Creatine kinase/s (0.6–6.7μkat/L)	85/85 (100)	30/31 (97)	40/40 (100)	31/32 (97)	34/35 (97)	220/223 (99)
TSH/s (0.27–4.2 mU/L)	83/85 (98)	31/31 (100)	38/40 (95)	30/32 (94)	33/35 (94)	215/223 (96)
Thriiodothyronin free/s (3.1–6.8 pmol/L)	84/85 (99)	31/31 (100)	40/40 (100)	32/32 (100)	36/36 (100)	223/224 (100)
Thyroxine free/s (12–22 pmol/L)	83/85 (98)	31/31 (100)	40/40 (100)	29/32 (91)	34/35 (97)	217/223 (97)
Angio-tensin converting enzyme/s (< 70 U/L)	81/83 (98)	31/31 (100)	38/40 (95)	32/32 (100)	36/36 (100)	218/222 (98)
Immunological assays—*no*. *POS/tested (%)*	* *	* *	* *	* *	* *	* *
Anti-nuclear antibodies (ANA/s,IgG)❡	14/85 (16)	2/31 (6)	5/40 (13)	2/32 (6)	4/36 (11)	27/224 (12)
Rheumatoid factor/s (> 20 IU/mL)	5/85 (6)	4/31 (13)	2/40 (5)	1/32 (3)	2/36 (6)	14/224 (6)
CCP-ab/s (IgG) (>7 IU/mL)	0 /85 (0)	0/31 (0)	0/40 (0)	1/32 (3)	1/36 (3)	2/224 (1)
HLA-typing/s (HLA DRB-1)	3/85 (4)	2/31 (6)	1/39 (3)	1/32 (3)	1/36 (3)	8/224 (4)
Myositis-ab/s (IgG)	19/85 (22)	7/31 (23)	7/40 (18)	8/32 (25)	4/36 (11)	45/224 (20)
CSF -analyses—*no*. *POS/tested (%)*	* *	* *	* *	* *	* *	* *
Cells LPK (total)/csf (> 5x10^6^/L)	2/67 (3)	0/27 (0)	2/36 (6)	0/23 (0)	0/27 (0)	4/180 (2)
Albumin/csf (>320 mg/L)	7/67 (10)	3/27 (11)	8/36 (22)	2/23 (9)	3/27 (11)	23/180 (13)
Lactate/csf (>2.5 mmol/L),	0/66 (0)	0/27 (0)	0/36 (0)	0/23 (0)	0/27 (0)	0/180 (0)
Glucose ratio/csf (<0.45)	0/67 (0)	1/27 (4)	2/36 (6)	1/23 (4)	1/27 (4)	5/180 (3)
IgG index (>0.63)	3/67 (4)	0/27 (0)	1/36 (3)	0/23 (0)	1/27 (4)	5/180 (3)
GFAp/csf	3/66 (5)	0/27 (0)	2/36 (6)	0/23 (0)	0/27 (0)	5/179 (3)
CXCL-13/csf (> 7.8 ng/L)	3/67 (4)	0/27 (0)	2/35 (6)	1/21 (5)	0/27 (0)	6/177 (3)
Oligoclonal bands	16/66 (24)	3/27 (11)	5/36 (14)	1/23 (4)**	3/27 (11)**	28/179 (16)
Borrelia index/csf (>0.3)	11/67 (16)*	0/27 (0)	0/35 (0)	0/23 (0)	0/27 (0)	11/179 (6)
Rickettsia-DNA/csf	0/67 (0)	0/27 (0)	0/36 (0)	0/23 (0)	0/27 (0)	0/180 (0)
Microbiological assays—*no*. *POS/tested (%)*						
Borrelia-ab/s IgG/IgM (> 22 IU/mL)	60/85 (71)*	31/31 (100)*	39/40 (98)*	0/32 (0)	0/36 (0)	130/224 (58)
Borrelia-ab’s Western Blot IgG/IgM	50/85 (59)*	27/31 (87)*	33/40 (83)*	0/32 (0)	0/36 (0)	110/224 (49)
Anaplasma phagocytophilum-ab/s (IgG)	6/85 (7)	0/31 (0)	8/40 (20)*	13/32 (41)*	0/36 (0)	27/224 (12)
Bartonella henselae/quintana-ab/s (IgG)	6/85 (7)	0/31 (0)	5/40 (13)*	5/32 (16)*	0/36 (0)	16/224 (7)
Babesia-ab/s (IgG)	0/85 (0)	0/31 (0)	0/39 (0)	0/20 (0)	0/36 (0)	0/211 (0)
Rickettsia-ab/s IgG/IgM	30/85 (35)*	0/31 (0)	35/40 (88)*	22/32 (69)*	0/36 (0)	87/224 (39)
Candidatus Neoerlichia DNA/s	2/85 (2)	0/31 (0)	0/40 (10)	1/32 (3)	0/36 (0)	3/224 (1.3)
Borrelia IgG and 1 co-infection (IgG)	17/85 (20)	0/31 (0)	33/40 (83)*	0/32 (0)	0/36 (0)	50/224 (22)
Borrelia IgG and ≥2 co-infections (IgG)	2/85 (2)	0/31 (0)	7/40 (18)*	0/32 (0)	0/36 (0)	9/224 (4)
Borrelia IgG neg ≥2 co-infections (IgG)	0/85 (0)	0/31 (0)	0/40 (0)	9/32 (28)*	0/36 (0)	9/224 (4)

✫ Bb s.l. denotes *Borrelia burgdorferi* sensu latu, TBI tick borne infection, N total number, NR normal range, no. number, POS positive, CCP cyclic citrulinated peptide, CSF cersebrospinal fluid, GFAp glia fibrillary acidic protein (in relation to age), PTLDS post-treatment LD syndrome.

❡ includes analysis of antibodies to dsDNA, centrimere B, nucleosomes, Jo1,ribosomal P, 68-RNP, SS-A52, A60-SS, SS-B.

* Statistically significant difference at p<0.05 level compared to the groups with no *.

### Reported symptoms

Symptom duration of more than one year was reported in 190/224 (84%) patients, with a median of four years (range 1–40). Tick bite was recalled by 165/224 patients (74%). In the PTLDS group, 73/85 (86%) patients had previously been diagnosed with ECM, 13/85 (15%) were previously noted for neuroborreliosis and 3/85 (3.5%) had documented ACA. Overall reported median number of symptoms was 8 (range 2–18). Antibiotic treatment, usually doxycycline, for LD or TBI had been prescribed at least once for 176/224 patients (79%). A higher proportion of these patients was found in Group 0 (*p* <0.05) and Group 1. Treatment had sometimes been repeated up to six times, with different antibiotics. The median antibiotic treatment duration was 14 days (range 10–350). The majority had experienced a sense of improvement during antibiotic treatment that did not remain after treatment termination. Investigation outside Sweden, usually in Germany and often on the patient’s own initiative, were reported by 29/224 patients (13%) of which seventeen of these 29 patients (8%) had been treated with intravenous antibiotics. As a result of their illness, 71/224 (32%) were on sick leave or had chosen to reduce their working hours. Reported symptoms, and their distribution across general, musculoskeletal, neurological, neurocognitive, joint and other symptoms in the five different groups are summarized in Table 2. No statistically significant differences in presented symptoms between the groups were observed. Fatigue was reported by 157/224 (70%) patients, but sleep disturbances and decreased endurance were also common. Up to 178/224 (79%) reported one or more of any of the musculoskeletal symptoms listed in Table 2. Neurological symptoms were experienced by 184/224 (82%) patients, with even presentation across Group 0–4. Neurocognitive impairments were reported by 128/224 (57%) patients, of which reduced memory, impaired concentration and depression were reported by 25–27%. Joint pain, affecting all joints equally, was reported by 112/224 (50%) patients. Additional symptoms were present in 168/224 (75%) patients, most commonly gastrointestinal or cardiovascular manifestations. No objective physical findings were noted that supported ongoing TBI.

### Laboratory results

Table 3 shows laboratory details for the 224 patients. Differences between Group 0–4 were consistently very small, but some differences from the normal values were noted. 32/223 (14%) patients showed elevated serum cobalamin (above 700 pmol/L) as a result of dietary supplements. Moderately elevated fibrinogen levels between 4.3–5.9 g/L were demonstrated in 47/224 (21%) patients, usually with a concomitant normal CRP, but with an elevated sedimentation rate in 19/224 (9%) patients, mainly in Group 3 + 4 (13–14%, range 17–27 mm) compared to 9% in Group 0 (range 16–73 mm) and 3–5% (range 17-27mm) in Group 1 + 2. Anti-nuclear antibodies were detected in 27/224 (12%) patients. Of these, 21/27 showed a discrete nuclear homogeneous pattern, 2/27 nuclear dots and 1/27 a centromeric pattern, probable without clinical significance. 4/27 had strong reactivity that was considered of possible clinical significance, three of these in Group 0 and one in Group 4, three of which were homogeneous in pattern and one with nuclear dots. Elevated rheumatoid factor (>20 IU/L) was found in 14/224 (6%). Nine of these were in the range 22–47 and five between 72–277 IU/L. The latter were found predominantly in Group 1 and 4. The HLA DRB1 allele was detected in 8/224 (4%) patients with a slight predominance for Group 0 and 1. Myositis antibodies to any of the following antigens were found in 45/224 (20%) patients included in the assay: PL-7, SSA/Ro52, Mi-2 alpha, Mi-2beta, PM-cl75, PM-Scl100, Jo-1, Ku, PM-Scl75, SRP, TIF1gamma, SAE1, SAE1/SUMO1, Mi2, PL12. 21/224 (9.4%) patients had medium to high titers to the SSA/Ro52 (5/21), PM-Scl75 (4/21), SRP (3/21), PL-7 (3/21), Mi-2 alpha (2/21) SAE (2/21), Jo1 (1/21) and Ku (1/21) antigens. 180 (80%) patients underwent lumbar puncture, and four (2%) of them–two belonging to Group 0 and two to Group 2 –had more than five white blood cells (range 8–22 poly/mono) in the CSF. None of them had elevated sedimentation rate or C-reactive protein. One of the four had previously been treated for neuroborreliosis and had residual IgG antibodies in CSF. These four patients had moderately elevated levels of *Borrelia* antibodies in serum, most notably IgG; one also had antibodies to *B*. *henselae* and one IgG to *R*. *helvetica*. They had all previously been treated once or several times with doxycycline, and in one of the cases also phenoxymethylpenicillin. Slightly to moderately elevated albumin levels in the CSF, often as a result of barrier damage and minor bleeding at lumbar puncture, were found in 23/180 (13%) patients, with the highest numbers of patients in Group 2, in single cases also in combination with simultaneous presence of oligoclonal bands. Of the lumbar-punctured patients, normal values for cells, CXCL-13, IgG indices, lactate, and glucose ratio in the CSF were found in between 94–100%. Only 5/179 (3%) patients had moderately elevated levels of glia fibrillary acidic protein in relation to age. Suspicions of other medical conditions, such as ALS or MS, or patients with high levels of myositis antibodies were in some cases referred for further assessment to neurologists and rheumatologists, where appropriate, though no conclusive diagnoses were made. In total, these patients comprised a handful of individuals among the entire group and were equally distributed across all groups. *Borrelia* antibodies were detected in the CSF of 11/67 (16%) patients in Group 0, representing 11/179 (6%) of the lumbar-punctured patients. None of these findings was considered to reflect a current infection, hence infection treatment was not recommended.

A positive *Borrelia* serology was demonstrated in 130/224 (58%) patients, of which 110/130 (85%) showed positive specific reactions in Western blot, all interpreted as previous exposure of infection. *Anaplasma* antibodies were found in 27/224 (12%) patients, to *Bartonella* in 16/224 (7%) and to *Rickettsia* in 87/224 (39%). A positive *Borrelia* serology was demonstrated in 7/27 (26%) patients who were serologically positive for *Anaplasma*, in 11/16 (70%) of the *Bartonella* positive and 29/87 (33%) of the *Rickettsia* positive patients. In 3/224 patients (1.3%), DNA from *Candidatus* Neoehrlichia mikurensis was detected in the blood. Of patients with a positive *Borrelia* serology, 50/130 (38%) were serologically IgG-positive for an additional TBI and 9/130 (7%) for two other TBIs. There were no significant differences between the laboratory results summarized for each analysis between the groups. The only exception was significant differences of serological results as a result of the division into groups. After clinical and laboratory examination, 14/224 (6%) patients were recommended antibiotic treatment based on laboratory and clinical findings that made infection difficult to exclude, for example high antibody titers in serum or CSF, raised cell count, or PCR findings (neoerlichia) in combination with the absence of previous treatment attempts. Of the 14 receiving treatment, six were treated for LD, three for anaplasmosis, three for neoehrlichiosis, one for rickettsiosis and one for bartonellosis. The latter is not a tick-borne disease, but was included in the investigation panel. The treatment used was doxycycline tablet 200 mg orally daily for 14 days. Group 0, 1 and 2 each had two *Borrelia* cases treated with antibiotics, while Group 3 and 4 had no cases, though this did not constitute a significant difference (*p* = 0.18 by Chi square test).

### Quality of life estimation

**Fig 1:**
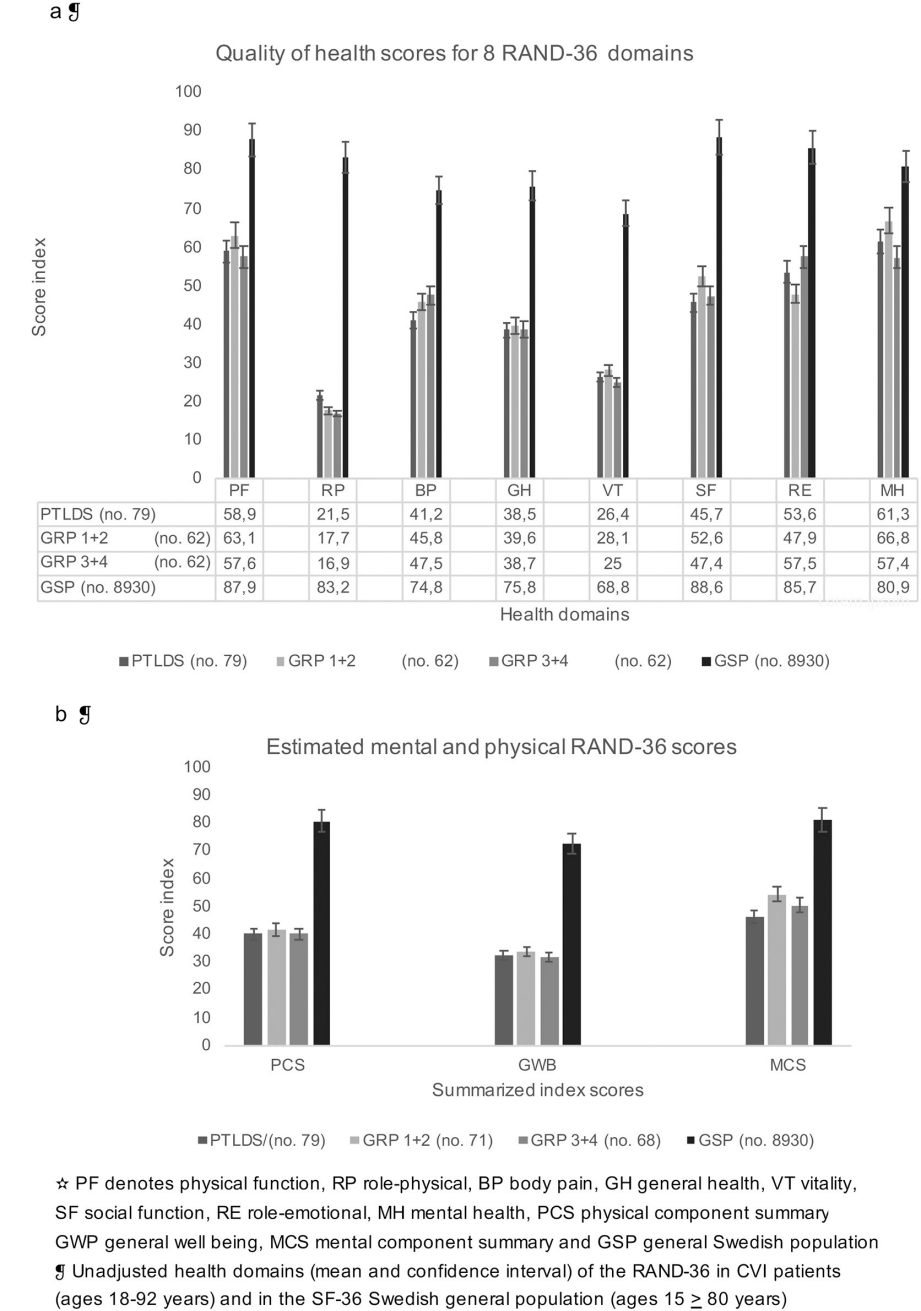
**a-b**-Quality of Life Estimation by RAND-36.

The RAND-36 item health survey is illustrated in Fig 1A and 1B and presents 205/224 patients’ assessment of their quality of life. All health domain scores for the CVI group, divided into Group 0, Group 1 + 2 and Group 3 + 4, showed significant statistical differences compared to the general Swedish population (z >3.29, α = 0.1%, Pr(>t) <0.001), but no significant difference was seen when comparing the individual eight health concepts between the Groups (*p* range 0.109–0.919).

## Discussion

In the present study, 224 adult Swedish patients with symptoms lasting for more than 6 months, including 85 patients who also fulfilled the criteria suggested for PTLDS, were investigated using a standardized clinical and laboratory panel. The results reveal no significant differences between the PTLDS group and the other subgroups with patients with similar persistent symptoms, but with or without exposure to TBIs. However, most patients referred to CVI were concerned that their symptoms were a consequence of a previous TBI. The clinical features, objective diagnostic criteria and appropriate treatment of patients with LD have been well described [24]. However, patients reporting persisting symptoms of unknown etiology, often following completion of conventional antimicrobial therapy, have caused a controversy regarding whether or not these manifestations are caused by persisting LD [24–26]. The diagnosis of CLD is often based solely on clinical assessments, in the absence of mandatory requirements for objective clinical or laboratory evidence of ongoing infection. A lack of antibodies to *Borrelia*–or as in Australia, a lack of both a LD vector and LD–has not prevented clinicians or patients from claiming that CLD exists, even when scientific evidence for seronegative chronic infection does not exist [27–30]. The study shows that symptoms often categorized as CLD in the general debate cannot be uniquely linked to LD or specifically to any of the other investigated TBIs. Regardless of whether previous exposure to one or more TBIs can be confirmed, patients testified to a similar set of symptom manifestations and also exhibited essentially similar, normal laboratory findings. The same was true of patients who matched the proposed case definition for PTLDS, and despite previously documented signs of LD, neither any particular symptoms nor distinguishing laboratory findings could be identified [10]. The majority of patients had undergone a single treatment, with a range of up to six treatments for LD for a period between 10 and 350 days and with at most four different antibiotics. The reason for treatment was usually the patient’s own request, thus treatment was not always based on clinical and/or supporting laboratory evidence for current infection. Apart from temporary improvements in some patients, the majority of symptoms remained afte ttreatment. The same was true of patients who had undergone prolonged, sometimes intravenous, antibiotic treatment outside Sweden. Previous randomized double-blind extended trials involving patients with persistent symptoms associated with LD have reported a similar lack of apparent improvement and no difference or advantage compared to short-term antibiotic treatment [31,32]. All patients in the study were carefully assessed for other medical conditions that might explain their symptoms. Of those 14 patients chosen for treatment, temporary improvement was observed in some patients, which was primarily judged to be due to psychological factors and the anti-inflammatory effects of doxycycline. Otherwise, the effect of the treatment was negligible in all but three patients with neoehrlichiosis, who at follow-up were PCR negative. The remaining 210 patients were, by consensus, not recommended for antibiotic treatment. It has been suggested that missed or delayed diagnosis and treatment of LD is a risk factor for development of PTLDS [33]. However, due to previous suspicion of LD, although the exact time of treatment was not always known, 82/85 (96%) patients in Group 0 and 94/139 (68%) in Group 1–4 had previously been treated with antibiotics. Myositis-specific antibodies were demonstrated in 45/224 (20%) of patients. A high proportion (21%) had slightly elevated fibrinogen levels, especially patients in Group 0 24%), anti-nuclear antibodies (12%) and elevated rheumatoid factor (RF) in 6% (range 3–13%), indicating an immunological/inflammatory reaction. However, the patients lacked objective symptoms associated with myositis, and the levels of creatine kinase were normal; hence no indication of muscle damage was seen. A possible methodological cause resulting in overdiagnosis must also be considered. However, in accordance with international recommendations from 2014, a cut-off limit is to be chosen for IF-ANA that gives a maximum of 5% positive in a healthy control group [34]. Methodological checks, including all 14 specificities, have been carried out regularly on healthy controls to confirm that the results are in accordance with the guidelines. Therefore, it can be assumed that findings of 6–16% (average 12%) positive in the present cohort represent a real increase compared to the prevalence of 5% in a healthy population. The same principle applies to RF when the classification criteria are followed, with a test designed such that no more than 5% positivity is found in a healthy control group [35]. In Sweden, one test is primarily used for myositis antibodies: Inflammatory Myopathies 16 antigen line immunoassay (Euroimmun, Lübeck, Germany). The Laboratory for Clinical Immunology in Uppsala, which performed the analyses, has reported 100% specificity for MSA and 98.7% for MAA, with a cut-off of 10 AU, when testing 60 healthy blood donors and diagnosed myositis controls participating in a quality assurance program. Previous reports have shown that, in addition to anti-SSA/Ro-52, anti-Ku and anti-PM/Sci, the antibody reactivities are quite myositis-specific, while the others are myositis-associated. In the present study, a total of 20/224 (9%) patients with MSA was found, of whom 12/224 (5%) showed high and medium levels. Limited data on the prevalence of myositis antibodies in healthy populations have been published, but MSA prevalence values up to 9% have been reported at a cut-off corresponding to the manufacturer’s recommended range [36,37]. These results are therefore difficult to interpret, but the presence of myositis antibodies in the study cohort is in a higher range, which together with the other findings may indicate an underlying inflammatory condition or represent a prodromal part/stage of an autoimmune rheumatic disease, an issue that needs to be studied further [38–40].

Studies of patients with CLD or PTLDS have consistently shown statistically significantly worse reported symptoms and quality of life among these patients than among controls from the general population [41,42]. The mean of the health concepts in Group 0–4, as measured by RAND-36, was significantly below that of the general Swedish population, especially regarding RP, VT, physical component summary and general well-being, findings consistent with those previously reported [41–43]. Most patients experienced fatigue, but other symptoms such as musculoskeletal pain, dizziness, depression and effects on cognitive functions were also common. The scores for the PTLDS group are comparable with those for the whole group.

## Conclusions

Consistency was seen in all respects among study participants, and no significant differences regarding symptoms, laboratory results or disease course were noted between patients who met the criteria for PTLDS or who had, or lacked, serologic evidence of exposure to *Borrelia* or other TBIs. The results do not support the notion that the symptoms are uniquely linked to Lyme disease. However, every fifth patient in the study showed signs of autoimmune reactivity, indicating that further studies, using a broad multifactorial approach, are needed to elucidate the underlying causes and mechanisms of PTLDS.

## Supporting information

S1 File Statistic test data and results Table 1. (XLSX)

S2 File Statistic test data and results Table 2. (XLSX)

S3 File Statistic test data and results Table 3. (XLSX)

S4 File Statistic test data and results Rand 36. (XLSX)
